# Editorial: Liver transplantation for liver cancer in the era of transplant oncology: accurate diagnosis and treatment

**DOI:** 10.3389/fimmu.2023.1276566

**Published:** 2023-09-11

**Authors:** Ze Xiang, Chiyu He, Di Lu, Shusen Zheng, Xiao Xu

**Affiliations:** ^1^Zhejiang University School of Medicine, Hangzhou, China; ^2^Key Laboratory of Integrated Oncology and Intelligent Medicine of Zhejiang Province, Hangzhou, China; ^3^National Health Council (NHC), Key Laboratory of Combined Multi-Organ Transplantation, Hangzhou, China

**Keywords:** liver transplantation, liver cancer, transplant oncology, lung metastasis, immunotherapy

A decade after being proposed, liver transplant oncology, as a model of multidisciplinary integration, has become clear and distinctive (1). Liver transplantation (LT) for hepatocellular carcinoma (HCC) accounts for a different proportion in different countries and regions, and the proportion reaches up to 45% in China (2). In the absence of organs, the bottleneck problem common to the East and the West today is how to achieve refined stratification and precise treatment of recipients, thereby reducing recurrence and metastasis.

First of all, the metastasis and recurrence rate of HCC remains high although the selection criteria of LT for HCC were expanded and optimized in the past ten years (3). The metastasis site is mainly extrahepatic, and lung metastasis accounts for 40%-60%, which requires particular attention (4). Among the many factors that influence lung metastasis, tumor biology is the most important and concerned as clear as a bell. The previous study found that tumor necrosis factor receptor 2 can serve as a histological biomarker predicting post-LT lung metastasis of HCC (5). Recently, liquid biopsy based on multi-omics enables screening of key molecules (6), and the visualization of these molecules will be greatly promoted with the development of artificial intelligence, machine learning and deep learning, so as to achieve clinical validation and application. Furthermore, factors other than tumor biology can also contribute to tumor metastasis and recurrence. The imbalance of metabolism and microbiology in liver transplant recipients will increase the risk of complications, mainly including biliary and vascular complications and liver failure. The gut-liver axis can interact with various organs and different systems, and its dysfunction will also aggravate the biological characteristics of malignant tumors, thereby causing metastasis and recurrence (7). In addition, as the soil for tumor cell seeding, the inflammatory microenvironment in the lung should be also further investigated, and its disorder under the treatment of immunosuppressive agents might be the key event mediating lung metastasis. Globally, it is of great significance to carry out multi-center clinical studies on post-LT lung metastasis, whether it is a large-sample study on the prevention and treatment, or a novel immunosuppressive scheme study.

The emergence of novel modalities allows new attempts in prevention and treatment of tumor recurrence after LT. Although existing immunotherapies have shown great therapeutic effects in the treatment of HCC, their use in LT is limited due to the lack of a standardized scheme and the risk of post-LT rejection (8). The safety should be fully considered, and it will be absolutely helpful to develop new anti-tumor immunotherapies that does not increase rejection risk (9). On the other side, the combined use of immunotherapies and other therapies has been a trend in clinic, including pre-operative downstaging and post-operative adjuvant therapies. The efficacy needs the support of evidence-based medicine. Therefore, more efforts should be made to develop novel immunotherapies. The application of chimeric antigen receptor-T (Car-T) therapies in LT for HCC is of exploratory significance (10). The use of Car-T therapies in immunosuppressive environment shows promising prospects.

In general, we have made great progress in the first decade of liver transplant oncology, while there also exist emerging challenges and turns. Multi-disciplinary cooperations are urgently needed to overcome these challenges in the near future.

## Author contributions

ZX: Writing – original draft. CH: Writing – original draft. DL: Writing – original draft. SZ: Writing – review & editing. XX: Writing – review & editing.

## References

[1] Liver EAfTSot. Easl clinical practice guidelines: liver transplantation. J Hepatol (2016) 64(2):433–85. doi: 10.1016/j.jhep.2015.10.006 26597456

[2] Chen J Shen T Li J Ling S Yang Z Wang G. Clinical practice guideline on liver transplantation for hepatocellular carcinoma in China (2021 Edition). Chin Med J (2022) 135(24):2911–3. doi: 10.1097/CM9.0000000000002515 PMC10106222 36602405

[3] Thuluvath PJ To C Amjad W. Role of locoregional therapies in patients with hepatocellular cancer awaiting liver transplantation. Off J Am Coll Gastroenterology ACG (2021) 116(1):57–67. doi: 10.14309/ajg.0000000000000999 33110015

[4] Sapisochin G Bruix J. Liver transplantation for hepatocellular carcinoma: outcomes and novel surgical approaches. Nat Rev Gastroenterol Hepatol (2017) 14(4):203–17. doi: 10.1038/nrgastro.2016.193 28053342

[5] Li H Lin Z Zhuo J Yang M Shen W Hu Z. Tnfr2 is a potent prognostic biomarker for post-transplant lung metastasis in patients with hepatocellular carcinoma. Chin J Cancer Res (2023) 35(1):66. doi: 10.21147/j.issn.1000-9604.2023.01.07 36910852 PMC9992998

[6] Ye Q Ling S Zheng S Xu X. Liquid biopsy in hepatocellular carcinoma: circulating tumor cells and circulating tumor DNA. Mol Cancer (2019) 18:1–13. doi: 10.1186/s12943-019-1043-x 31269959 PMC6607541

[7] Xiang Z Wu J Li J Zheng S Wei X Xu X. Gut microbiota modulation: A viable strategy to address medical needs in hepatocellular carcinoma and liver transplantation. Engineering (2023). doi: 10.1016/j.eng.2022.12.012

[8] Gu Y Xu S Wang Z Yang J Zheng S Wei Q. When immunotherapy meets liver transplantation for hepatocellular carcinoma: A bumpy but promising road. Chin J Cancer Res (2023) 35(2):92. doi: 10.21147/j.issn.1000-9604.2023.02.02 37180832 PMC10167603

[9] Hu Z Yang M Chen H He C Lin Z Yang X. Double-negative T cells: A promising avenue of adoptive cell therapy in transplant oncology. J Zhejiang University-Sci B (2023) 24(5):387–96. doi: 10.1631/jzus.B2200528 PMC10186140 37190888

[10] Sterner RC Sterner RM. Car-T cell therapy: current limitations and potential strategies. Blood Cancer J (2021) 11(4):69. doi: 10.1038/s41408-021-00459-7 33824268 PMC8024391

